# A window of opportunity? The relevance of the rotating European Union presidency in the public eye

**DOI:** 10.1177/14651165221142504

**Published:** 2022-12-04

**Authors:** Olga Eisele, Tobias Heidenreich, Nina Kriegler, Pamina Syed Ali, Hajo G. Boomgaarden

**Affiliations:** Department of Communication, 27258 University of Vienna, Austria; Department of Communication, University of Amsterdam, The Netherlands; 28422 Berlin Social Science Centre, Germany; Department of Communication, 27258 University of Vienna, Austria; Department of Communication and Media Research, 27217 University of Zurich, Switzerland; Department of Communication, 27258 University of Vienna, Austria

**Keywords:** Austria, automated content analysis, manual content analysis, media coverage, rotating EU presidency

## Abstract

The rotating EU presidency's relevance for EU politics has decreased since the introduction of a permanent council president. However, news salience and framing of the own government acting as the EU presidency can amplify publicity for EU affairs. We, therefore, evaluate the visibility and framing of the EU presidency in 12 Austrian newspapers for 2009–2019. We conduct an automated text analysis of 22 presidencies over 11 years, testing several hypotheses statistically, and qualify results via manually coded frames of the Austrian EU presidency in 2018. The results confirm the crucial importance of the domestication of EU politics, underscoring the potential of the presidency to serve as a window of opportunity for public debate. We discuss our findings with reference to the EU's democratic deficit.

## A window of opportunity

With the Lisbon Treaty, the institution of the rotating European Union (EU) presidency of the Council of the EU (Council), held by individual member states in turn for six months respectively, was partly substituted by the permanent president of the European Council (EuCo). This innovation is judged to be an important improvement, providing continuity and consistency since the EuCo, the institution in which EU member states’ heads of state and/or government meet regularly, is ‘no longer subject to national grandstanding, occasional weak leadership and uneven presidential performance’ (Dinan, 2013: 1256). In light of this development, the political relevance of the rotating presidency has decreased. Its tasks have, ever since the entering into force of the Lisbon Treaty, been confined to the Council – the EU body constituted by several formations representing the different political portfolios, for example, the Council of Finance Ministers or the Council of Ministers for Agriculture and Fisheries. Here, the rotating presidency helps to ensure the smooth operation of the Council's everyday political work; Trio presidencies (so-called trios), three member states holding the presidency consecutively, shape the larger-scale common agenda of the presidency while each member state also defines its own priorities (General Secretariat of the Council, 2020).

We argue that the fact that a national government is chairing the EU is relevant for EU politics in a different way, namely by evoking publicity for the EU in the respective country, thereby helping to somewhat alleviate its notorious democratic deficit. Public debate is crucial to empower citizens in a democracy, providing them with the information needed in order to evaluate politics and hold politicians accountable (Habermas and Cronin, 2012: 49). However, due to its remoteness and complexity, EU politics did for long not seem particularly newsworthy while, owing to a lack of first-hand experience, news coverage about the EU is often the only source of information for citizens (Marquart et al., 2018). More than a decade of crises has increased the new prominence of the EU (e.g. Boomgaarden and De Vreese, 2016). But the coverage has also become more negative (e.g. Marquart et al., 2018) in a climate of increasing Euroscepticism and, more generally, political disenfranchisement and distrust (Baglioni and Hurrelmann, 2016; Dotti Sani and Magistro, 2016). An EU presidency held by an individual member state has a stronger connection to its citizens and might therefore present a window of opportunity for public discourse. Also, against the background that EU news have been found to most often exhibit a national angle (e.g. Eisele, 2017; Gattermann, 2013), the presidency of the own government could help to make EU politics more visible (Boomgaarden et al., 2013), empowering citizens to better inform themselves. Potentially, it could also increase EU legitimacy by conveying an image of the political relevance of the rotating presidency and EU politics as such, thereby motivating citizens to get involved (for a more general argument see, for instance, Moon, 2013).

The academic literature on EU presidencies has mostly neglected the issue of publicity so far and has generally been described as repetitive (Schout, 2017). Indeed, it is overwhelmingly focused on evaluating the performances of single presidencies (e.g. Bilčík, 2017; Fuchs, 2020; Karolewski et al., 2015; Schout, 2017), in some cases in a more comparative manner (Batory and Puetter, 2013; Schout and Vanhoonacker, 2006; Vaznonytė, 2020). Some contributions develop general frameworks for evaluating the success of a presidency (Häge, 2017; Smeets and Vennix, 2014; Toneva-Metodieva, 2020; Vandecasteele and Bossuyt, 2014) or embed the presidency in integration theories (Coman, 2020; Puetter, 2014). Research has investigated the role of national parliaments during EU presidencies (Kaniok and Nováková, 2021), or addressed the influence of the presidency on institutional power relations more generally (Van Gruisen et al., 2019; Van Gruisen and Crombez, 2021). Analyses of how EU presidencies are discussed in the public sphere, however, are scarce (see Stepinska, 2014). We, therefore, ask whether and how the EU presidency indeed affects public debate about the EU as represented in media coverage, potentially allowing for a more informed and engaged citizenry.

We analyse the media salience and the framing of 22 EU presidencies in eleven years of Austrian print news (2009–2019). Rooted in the theoretical discussion about newsworthiness and news values, we selected Austria as a small EU member state with a somewhat volatile relationship with the EU. Austria is often described as a bridge-builder and reliable partner in EU politics. However, the chancellery of Sebastian Kurz and the coalition of his Austrian People's Party (ÖVP) with the Austrian Freedom Party (FPÖ) cast some shadows on Austria's relationship with the EU as the rather EU-critical stance of the FPÖ was more and more taken up by the ÖVP, also shaping the EU presidency in 2018 (Heinisch et al., 2020). Our dataset was originally collected for another project; it includes 12 Austrian print newspapers representing different journalistic styles, published at the national and regional level, covering a short period before, but mostly the time after the introduction of the permanent EuCo president. To understand and explain how the presidency may serve as a window of opportunity, we analyse 11 years of news content drawing on computational approaches, including Latent Semantic Scaling (LSX; see Watanabe, 2021), and test several hypotheses in a regression analysis. To gain a deeper understanding of the quality of presidency news, we then zoom into coverage of the Austrian presidency, relying on a focussed manual content analysis of media frames in the second half of 2018. We conclude by discussing our results considering the EU's legitimacy deficit.

## EU presidencies in the news

The decision on how journalists use the scarce news space available to them and decide which information is eventually published is based on an evaluation of newsworthiness. The literature has repeatedly produced lists of news values that characterise published news items. Even if such accounts ultimately hinge upon how the published story was constructed by the journalist rather than defining any sort of a priori objective characteristics, they can still help in approaching the question of what might increase salience. *Salience* is an important category in political media effects because it structures what issues news audiences deem important or what yardsticks they resort to when forming political opinions (e.g. Moy et al., 2016). The news agenda is generally a strong predictor of citizens’ political attitudes (e.g. Damstra et al., 2021). A news agenda featuring EU politics, and the presidency, in particular, would signal to citizens that this is a relevant topic to think about and form an opinion about. Going beyond the mere visibility of presidencies, the way how issues are covered – how they are *framed* – is regularly found to be an important factor regarding the effects of news coverage on public opinion, also regarding EU affairs (Marquart et al., 2018). Framing as such refers to the process of ‘select[ing] some aspects of a perceived reality and mak[ing] them more salient in a communicating text, in such a way as to promote a particular problem definition, causal interpretation, moral evaluation, and/or treatment recommendation for the item described’ (Entman, 1993: 52). Media framing influences how people think about the issue in question and is, therefore, a crucial factor for explaining citizen's political attitudes and engagement, (e.g. Camaj, 2014; Moon, 2013).

### The salience of the rotating EU presidency in Austrian media coverage

Media *salience* of the EU presidency, that is, its visibility in news media coverage, may be read as an indicator of a news item has fulfilled the standards of newsworthiness. The *domestication* and *proximity* of news are found to be important news values overall (Harcup and O’Neill, 2017), and for EU news in particular, a national angle as well as geographical proximity is found to prevail (Eisele, 2017; Heidenreich et al., 2022; Walter, 2016). We, therefore, expect presidency coverage to be shaped by these news values as well.

#### Domestication

EU news is repeatedly found to promote a national, domestic angle on EU politics rather than a genuinely European perspective (Dutceac Segesten and Bossetta, 2019; Gattermann, 2013) and to attribute more newsworthiness to national aspects of EU politics (Eisele, 2017). We, therefore, assume the rotating EU presidency to be more visible in Austrian media coverage in the second half of 2018 when it was held by Austria.

*H1a:* During the presidency of the national government, the EU presidency will be more salient in national print news.

#### Trio presidency

In addition, the trios are constituted of three consecutive presidencies which, together, prepare a programme spanning all three presidencies while each presidency formulates its own goals as well (General Secretariat of the Council, 2020). Austria committed to this task together with Estonia (July–December 2017) and Bulgaria (January–June 2018). Austria may, therefore, already become visible in the national media regarding its upcoming presidency and the goals formulated together with the Estonian and Bulgarian presidencies, thus from the second half of 2017 onwards. The common Trio presidency is also assumed to make the participating presidencies more dependent on each other; thus, possible positive or negative influences of the Trio partners may also play a role in increasing the news visibility of EU presidencies overall during that specific period.

*H1b:* During the period of the Trio presidency which the national government is part of, the EU presidency will be more salient in national print news.

#### Proximity

Greater geographical proximity of the country holding the presidency, that is, a neighbouring country, is assumed to increase relevance to the audience as it indicates closer cultural and political ties due to, for example, a common language or common borders entailing greater interdependence (for a similar argument see Walter, 2016).

*H1c:* The greater the geographical proximity of a member state holding the EU presidency to the country in which the newspapers are published, the more salient the EU presidency is in national print news.

Moreover, we expect that member states with greater *power* in the EU will be regarded as more newsworthy by journalists, as they can induce change more effectively, also against the opposition, and shape EU politics for all other member states as well (e.g. Müller and van Esch, 2020; more generally Weber, 1978: 53). Drawing on research evaluating EU presidencies and the general literature debating power division lines in the decision-making processes within the EU (e.g. Archer and Nugent, 2006; Degner and Leuffen, 2019), this may concern political weight, financial power, or political experience.

#### Political weight

The size of a member state in terms of population is institutionalised in the EU's polity, for example as the basis for calculating the seat shares in the European Parliament allocated to a member state or the voting weights in the Council in such policy fields where member states do not agree unanimously (e.g. General Secretariat of the Council of the European Union, 2021). Larger member states also tend to be more powerful in economic terms, giving them additional weight in negotiations (see, for instance, Degner and Leuffen, 2019). Such member states are advantaged and sought-after coalition partners that can shift balances in negotiations (e.g. Archer and Nugent, 2006). Overall, the EU's institutional structure gives larger member states generally more influence in the EU's decision-making process, which we expect to increase their news value for journalists.

*H2a:* The more politically weightful the member state holding the EU presidency, the more salient the EU presidency is in national print news.

#### Financial power

Member states that can build on a large budget for their presidency are advantaged in their organisation as it is usually rather expensive, including also large publicity-boosting, potentially newsworthy, events at which the presidency can show off its national assets (Schout, 2017; Stavridis and Giannou, 2019). Financially more powerful member states could, therefore, be more successful in catching journalists’ attention.

*H2b:* The more financially powerful the member state holding the EU presidency, the more salient the EU presidency is in national print news.

#### Organisational experience

Regarding the organisation of the presidency in particular, a high degree of experience in terms of expertise, credibility, and reputation has been discussed as crucial. This concerns especially ‘older’ member states due to their implied greater organisational capacities and resources as well as their informal networks and insider knowledge about ‘how things work’ in the EU (Quaglia and Moxon-Browne, 2006; Schout, 2017; Vandecasteele and Bossuyt, 2014).

*H2c:* The more organisationally experienced the member state holding the EU presidency, the more salient the EU presidency is in national print news.

Finally, the news value of *conflict* is repeatedly found to be a crucial predictor of news coverage, also about the EU (Auel et al., 2018; Harcup and O’Neill, 2017; Van der Pas and Vliegenthart, 2016). We expect that greater (potential for) conflict will increase the newsworthiness of an EU presidency as it may be an indicator of political failure or struggle, that is, irrelevance regarding the presidency's aims and progress.

#### Elite conflict

The (positive) EU attitude of the governments holding the presidency has been described as one factor influencing if the presidency can politically make a difference or not (Quaglia and Moxon-Brown, 2006). Regarding news values, in contrast, the positive influence of elite polarisation and conflicts over EU issues on news visibility has been discussed in many studies analysing EU media coverage (Auel et al., 2018; Boomgaarden et al., 2013; Gattermann, 2013). This can be expressed as a potential conflict with the EU level, that is, an EU-critical government (Heinisch et al., 2020), or the potential for contesting EU politics in the national parliament (Auel et al., 2018).

*H3a:* The more EU-critical the stance of the government holding the EU presidency, the more salient the EU presidency is in national print news.
*H3b:* The greater the share of EU-critical parties in the parliament of the member state holding the EU presidency, the more salient the EU presidency is in national print news.

#### Public contestation

In addition, public opinion about the EU may differ from the government's stance, causing dissonance regarding, for example, protests during public events organised during the presidency. This might increase the news value of such events in terms of conflict and negativity (Boomgaarden et al., 2013; Harcup and O’Neill, 2017).

*H3c:* The lower public support for the EU in the member state holding the EU presidency, the more salient the EU presidency is in national print news.

### The framing of the rotating EU presidency in Austrian media coverage

EU politics has long not been regarded as particularly relevant and is generally found to suffer from a communication and participation deficit (e.g. Boomgaarden et al., 2013). While it has gained in public salience due to several accumulating crises, this greater visibility has been accompanied by increased negativity in coverage and a rise of public Euroscepticism (e.g. Marquart et al., 2018). Against this background, it is important to understand how the presidency is framed in terms of political relevance, defined here as ‘the degree to which something is related or useful to what is happening or being talked about’ (Cambridge English Dictionary, 2020). We, therefore, also explore how far the EU's rotating presidency is publicly ascribed to be politically related and useful for (EU) politics, in the sense of politically succeeding in conducting the tasks ascribed to the presidency.

The literature evaluating the relevance of presidencies in institutional terms lists concrete attributes of political relevance: Here, a presidency of political relevance, that is, one that has proved politically useful, is rooted in the holding member state's high degree of expertise, credibility, reputation, organisation, and the general attitudes of the government towards EU integration. Other factors highlighted are contextual, such as the general political conditions and circumstances of presidencies at the national or the EU level, for example, if the presidency is held during a crisis period coming with increased political and public pressure to find solutions (Quaglia and Moxon-Browne, 2006; Schout, 2017; Vandecasteele and Bossuyt, 2014). Apart from these more general considerations, however, it is hard to derive any specific expectations on the media framing of the presidency given the scarce literature on the news prominence of EU presidencies and on the aspect of relevance framing in particular. This part of our analysis is, therefore, exploratory. To keep analyses comparable, we rely on the same factors identified for our analysis of salience, namely proximity, influence, and conflict.

## Data and methods

The order of countries holding the presidency of the Council for our period of analysis was detailed in the Council's decision of 1 December 2009 (2009/208/EU) (see Table 1). It does not include the traditional political heavyweights in the EU, Germany, and France (e.g. Crespy and Schmidt, 2014). It does, however, include countries with somewhat strained relationships with the EU or at least a difficult political position during the time of their presidency. Ireland, Greece, and Italy were all holding the presidency in the wake of the financial crisis which had affected them much more than other members. The presidencies of the Czech Republic and Hungary, and more recently Romania and Bulgaria, were held under conflictual conditions regarding the EU's evaluation of domestic political developments, which, however, did not seem to constrain the success of their terms considerably (Coman, 2020: 587–589).

**Table 1.** Rotating presidencies of the council of the EU, 2009–2019.

Year	January–June	July-December
2009	Czech Republic	Sweden
2010	Spain	Belgium
2011	Hungary	Poland
2012	Denmark	Cyprus
2013	Ireland	Lithuania
2014	Greece	Italy
2015	Latvia	Luxemburg
2016	Netherlands	Slovakia
2017	Malta	Estonia
2018	Bulgaria	**Austria**
2019	Romania	Finland

*Note:* The permanent presidency of the EuCo was introduced with the Lisbon treaty in 2009 and took effect at the beginning of 2010.

Regarding our case study Austria, the conflicts induced by a decade of EU crises resulted in heated domestic debates and also changes in the Austrian political system: Being long governed by a grand coalition headed by Chancellors of the Austrian Social Democratic Party (SPÖ), the ÖVP formed a coalition with the right-wing populist FPÖ after general elections in 2017 and was in power also for the time of the Austrian EU presidency in the second half of 2018 (Heinisch et al., 2020; see Table 1). Shattered by a large-scale political scandal, this coalition broke in May 2019 and was followed by a caretaker government ruling until the end of our period of analysis (Auel and Pollak, 2019). The increasingly more EU-critical climate represented by Austrian policymakers seems echoed in the priorities of the Austrian EU presidency which emphasised the principle of subsidiarity while, however, at the same time also defining its task as a neutral bridge builder, eager to contribute to the EU's unity (Austrian Federal Chancellery, 2018).

### Eleven years of Austrian media coverage

To test our hypotheses and answer our research question, we analyse eleven years of Austrian media coverage of the rotating EU presidency (2009–2019, *n* = 11,098). This set of articles is drawn from a larger dataset of coverage about the EU in general,^1^ consisting of 12 daily tabloid and broadsheet newspapers published in Austria at the national and regional level (see, e.g. Greussing and Boomgaarden, 2017 for a similar strategy; see Table 2). Print news in general, while declining in popularity, was still a well-read news source in our period of analysis, ranking third behind TV and online news sources. Many newspapers in the sample, however, saw a considerable increase in digital subscriptions. In Austria, many newspapers share the same newsroom with their digital editions, with partly overlapping coverage online and offline. While the leading tabloid newspaper *Kronenzeitung* has by far the largest outreach compared to other newspapers (see Table 2), the broadsheets included, that is, *Standard* and *Presse*, belong to the most trusted news sources in Austria (see Sparviero and Trappel, 2021).

**Table 2.** Newspapers included in the sample.

Newspaper	Type	Market share
Kronenzeitung	National tabloid	23.9%
Heute	National tabloid	8.8%
Österreich	National tabloid	6.7%
Kurier	National broadsheet	6.5%
Standard	National broadsheet	7.3%
Presse	National broadsheet	4.1%
Kleine Zeitung	Regional broadsheet	9.9%
Oberösterreichische Nachrichten	Regional broadsheet	5.2%
Tiroler Tageszeitung	Regional broadsheet	3.3%
Salzburger Nachrichten	Regional broadsheet	2.8%
Vorarlberger Nachrichten	Regional broadsheet	2.1%
Wiener Zeitung	Regional broadsheet	No info

*Note:* Market share variable is taken from Verein ARGE Media-Analysen (2021).

For subsetting the data to include only articles discussing the EU presidencies, we developed a search string including relevant terms defining the visibility of the rotating EU presidency in EU coverage. Starting from the key term ‘Ratspräsidentschaft’, we inductively identified other relevant terms based on a sample of 850 articles, resulting in a search string to identify documents related to the EU presidency (see the Online appendix).

### Automated content analysis

The *salience* of the rotating presidency is operationalised as the visibility of the presidency and was defined based on our search string (see above). For the evaluation of the presidencies’ relevance, we made use of the document scaling technique *Latent Semantic Scaling* (LSS), developed by Watanabe (2021) and implemented via the R package LSX. In that sense, the automated analysis does not provide the same holistic view as manual coding but sheds light on the evaluation of the presidency in terms of relevance over time.

Combining dictionary analysis (i.e. the selection of model terms defining a ‘topic’) with word embeddings (i.e. polarity scaling) allows for scaling documents on different dimensions regarding a defined polarity. An example would be the positive or negative sentiment (polarity) of migration (topic) coverage or, as in our case, the political relevance or irrelevance (polarity) of EU presidencies (topic), defined via our search string.

Seed words for this approach should be unambiguous to a topic, have a strong polarity, and be corpus-independent. To define such seed words, we drew on the manual coding (see also above); we additionally collected markers based on which the variable ‘relevance’ was coded (for more information see the Online appendix). We also checked the actual mention of seed words in the corpus to assure that they can serve as markers for the relevance polarity. The selection of seed words (see Table 3) was validated on a fresh sample of EU presidency-related articles (*n* = 500) by two trained coders, who reached a satisfactory intercoder-reliability level (Percentage agreement = 0.82). Following similar validation approaches (Heidenreich et al., 2022; Trubowitz and Watanabe, 2021), we compared the mean scores of the articles for three-month windows to assess the accuracy of the model. With a correlation coefficient of *r* = .83, the LSS model was deemed useful to examine the relevance of the EU presidency in the texts. For more information on the approach, for instance, for coding instructions and examples from the automated coding, please see the Online appendix.

**Table 3.** Seed words for political relevance and irrelevance.

	DE original for analysis	EN translation
Relevant	Einstimmig, fortschritt, geschafft, höhepunkt, positiv, stark	Unanimous, progress, accomplished, highlight, positive, strong
Irrelevant	Absage, empörung, enttäuscht, kritisiert, negativ, reinfall	Cancellation, indignation, disappointed, criticised, negative, failure

### Regression analysis

Two models were fitted for the whole period of analysis (2009–2019), for the following *dependent variables*: (1) The salience of the EU presidency, which we operationalise as the share of articles addressing the EU presidency in EU coverage. Using a relative measure instead of, for example, the absolute number of articles, makes our analysis somewhat less sensitive to the influence of external shocks such as the migration crisis, and allows tailoring the analysis to the specific topic of the presidency as such. (2) The evaluation of the presidency in terms of political relevance for the EU. The variable contains the continuous scaling as obtained from the automated content analysis (see above).

Regarding the *independent variables*, we identified the country holding the presidency during the respective period to understand the influence of domestication and proximity; We added Austria (*H1a*) as well as Estonia and Bulgaria (*H1b*) to account for the period of the Trio presidency. For each country, we determined the proximity from Austria to the country holding the EU presidency during the respective period (*H1c*), operationalised as the geographical distance between capitals in kilometres (see Trilling et al., 2017 for a similar approach). For approximating member states’ power (*H2a*), we relied on the population size of the country which is the basis of the voting procedures of the Council (see General Secretariat of the Council, 2021). The country's GDP per capita served to operationalise financial power^2^ (*H2b*); the experience (*H2c*) of the member state holding the presidency regarding EU politics in general and the organisation of the presidency was included as the number of presidencies organised before (see 2009/208/EU). For determining the EU position of a government (*H3a*) and the share of EU-critical parties in parliament (*H3b*), we relied on the Chapel Hill Expert Survey trend dataset (Bakker et al., 2020). Both scores are weighted by the share of the respective parties in the parliament/government; we selected the values of the survey conducted closest to the year of the presidency. Public support for the EU (*H3c*) was operationalised using the survey item ‘Trust in the EU’ (share of positive replies) of the Standard Eurobarometer Surveys fielded during the respective presidency. Please refer to the Online appendix for an overview of independent variables and variation across countries.

Both models were fitted using linear regression and checked for multicollinearity issues (see the Online appendix). All variables were included in their standardised form (*M* = 0, *SD* = 1). In addition, we added several *control variables*, such as the level of publication of the newspaper (national or regional). We added the journalistic routine (tabloid or broadsheet) as such routines generally offer the most compelling explanations for differences in how newspapers cover politics (Jandura and Friedrich, 2014; see the Online appendix for regression models including fixed effects of individual newspapers). Lastly, we also controlled for the year 2009 when the permanent Council president was not yet institutionalised, and the newsworthiness and political relevance of the rotating presidency is assumed to have been greater.

### Manual content analysis

For the manual content analysis of coverage of the Austrian EU presidency, we inductively developed a coding scheme based on a random sample of articles mentioning the key term ‘council presidency’ (‘Ratspräsidentschaft’), to account for the frame dimensions that newspapers may use when mentioning the presidency (see Table 4). Coverage about the presidency was not found to be very rich, and often the presidency was only briefly described in an article discussing other political issues. We, therefore, developed a rather broad coding scheme to avoid increased granularity and fuzziness of results. We coded the frame dimension (on the label ‘dimension’, see Boydstun et al., 2013; see Table 4 for an overview of dimensions), thus the aspects made more salient about the presidency (Entman, 1993). These dimensions are then joined with the automated analysis of relevance (see the following section) as an evaluation of the presidency to provide a holistic news frame for the rotating EU presidency.

**Table 4.** Frame dimensions in Austrian EU presidency news coverage.

Dimension	Description and examples	Rel.
**Political work**	Focus on political work of the presidency, for example, agenda, outcomes of summits, negotiations on behalf of the EU, priorities	*0.67*
*Conservative and liberal European politicians have voiced massive criticism of the Austrian EU Council presidency under Chancellor Sebastian Kurz (ÖVP) in interviews, according to an advance report in the news magazine "profil." The criticism was particularly ignited by Austria's withdrawal from the UN migration pact during the presidency.*		
**Interior Politics**	Focus on how interior politics might/does influence the EU presidency, e.g., potential conflicts with EU critical government party FPÖ	*0.90*
*Benjamin Abtan of the anti-racist initiative Egam thinks that Austrian Minister of the Interior, Herbert Kickl, wanted to use an appearance at the EU event as a ‘fig leaf’ to cover up his own xenophobic policies. ‘Two things were unusual from the start: that we were meeting outside Brussels and that the logo of the Austrian presidency was on the invitation’, Abtan tells STANDARD.*		
**Public relations**	Focus on public events and their organisation during the presidency, for example, increased border controls before summits, police corps workload, sponsors of public events, locations, catering, programme	*0.90*
*Although Schloss Hof in Marchfeld overlooks numerous fields, the sophisticated fortress has hardly housed farmers in the past. Here, between the petting zoo and baroque gardens, the EU agriculture ministers are currently meeting in the framework of the Austrian Council presidency.*		
**Reference**	Presidency only very briefly mentioned in passing as a reference date or attribute of a politician in an article on other issues	*0.67*
*The speaker of the Austrian parliament, Wolfgang Sobotka, again assured that the Parliament would be able to conduct two committees of inquiry at the same time despite the EU Council Presidency. Parliamentary Director Harald Dossi stressed that the draft budget had been prepared accordingly.*		

*Note:* Reliability was measured based on a sample of 250 articles coded by two trained coders. Reliability is indicated in Percentage Agreement based on dummy variables indicating the presence of a frame dimension in an article. Agreement regarding the topic of the article being Austrian presidency was at 0.9. Lower scores in *Reference* and *Political Work* mainly stem from the difficulty of distinguishing between the two, that is, to determine if the presidency was only briefly mentioned as, for example, a reference date, or if the article was already more elaborate.

We only coded one dimension per article, that is, the most dominant focus, as our preliminary analysis found coverage about the presidency not to be very elaborate in general. Assuming that coverage would start already before and not end directly after the term, the coding scheme was used to code one year (April 2018–March 2019) of Austrian presidency coverage of all newspapers included in the analysis, situating the Austrian EU presidency (July 2018–December 2018) in the middle of this period. We only coded articles when they discussed the Austrian presidency; Articles discussing other presidencies were coded as irrelevant and discarded for this analysis. The articles for our period of analysis amount to a total of *n* = 4046. We coded a random sample of around 50% (*n* = 2050). These articles were coded by two trained coders and checked for inter-coder reliability (in percentage agreement; see Table 4). Considering the difficulty with reaching reliability in frames coding overall (# e.g. Matthes and Kohring, 2009), scores are deemed satisfactory.

## Results

**Figure 1.**
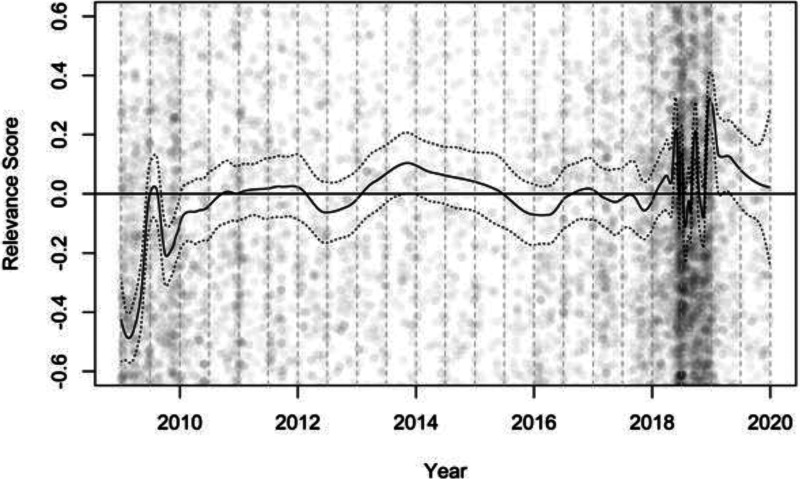
Salience and relevance of the rotating EU presidency in Austrian print news over time.

*Note:* Relevance scaling is smoothed using local weighted regression (loess); dotted lines are 95% confidence intervals. Salience is represented in the background density plot.

To analyse how the presidency of the own government affects media coverage, we studied the period of 2009–2019 to understand: (a) the salience of the EU presidency over time; and (b) the evaluation of presidencies as politically relevant or irrelevant (see Figure 1).

Starting with salience, the density of coverage (Figure 1) increases in 2018, especially in the second half. We also see a slightly increased volume of coverage for the year 2009, decreasing in 2010, when the permanent presidency took effect, and remaining low until 2017. Regarding the evaluation of presidencies in terms of political relevance, Austrian newspapers seem more critical of their ‘own’ government than of others, with, also due to greater salience, variable evaluations in 2018. For the first half of 2009, when the Czech Republic held the presidency, evaluations seem rather critical. Other than for these specific periods, the relevance of presidencies seems to be evaluated in a rather balanced way.

The results of this automated coding, moreover, were analysed using linear regression to identify factors explaining greater salience and an evaluation of the relevance of the presidency (see Table 5).

**Table 5.** Linear regression models for salience (DV1) and relevance (DV2)^3^.

	Model 1: Salience B (SE)	Model 2: Relevance B (SE)
*Hypothesis 1*		
Austria	0.654*** (0.023)	0.007 (0.038)
Trio presidency 2: Bulgaria	0.434*** (0.017)	0.040 (0.028)
Trio presidency 1: Estonia	0.074*** (0.019)	−0.008 (0.032)
Distance	0.034 (0.022)	−0.050 (0.039)
*Hypothesis 2*		
Population country	0.065** (0.021)	−0.018 (0.037)
GDP	0.001 (0.028)	0.088 + (0.050)
Presidencies before	−0.007 (0.029)	−0.090 + (0.051)
*Hypothesis 3*		
Government EU stance	−0.035 (0.026)	0.047 (0.044)
Share critical parties	−0.029 (0.020)	0.031 (0.035)
Trust in EU	0.063** (0.020)	−0.033 (0.035)
*Controls*		
Outlet type: broadsheet (vs. tabloid)	0.089*** (0.019)	0.008 (0.037)
Level: national (vs. regional)	0.031 (0.019)	−0.050 (0.031)
Year 2009	0.216*** (0.018)	−0.168*** (0.030)
R-Squared	0.609	0.031
*F-Statistic*	F (13,1501) = 182, *p* < .05	F (13,1204) = 4.08, *p* < .05
*N*	1515	1254^4^

*Note:* Coefficients are standardised B-coefficients, standard errors in parentheses. + *p* < .1, *p < .05, **p < .01, ***p < .001.

The salience of the presidency (Model 1, Table 5), measured as the share of presidency news in EU news, is increased for the Austrian EU presidency in 2018 and the two preceding presidencies (trios), other variables only have a small effect on visibility: Higher political weight and higher trust in the EU somewhat increase visibility; broadsheets provide more coverage about the presidency; and lastly, salience is increased in the year 2009 before the permanent presidency of the EuCo was introduced with the Lisbon Treaty. Regarding the evaluation as politically relevant or not (Model 2, Table 5), only the year 2009 seems to make a difference in that relevance evaluations were slightly more critical then.

Turning to the results of our manual content analysis, we investigated how the Austrian EU presidency is framed in Austrian print news, combining the manually coded frame dimension with the automated relevance coding for each article discussing the Austrian EU presidency between April 2018 and March 2019. In most articles, the presidency was framed in terms of *Political Work*, while the least discussed connection was made to *Interior Politics* (see Table 6). Regarding the evaluation of the Austrian presidency, coverage is overall rather balanced but also shows some volatility of opinions mirrored in the standard deviation.

**Table 6.** Descriptive statistics for frame dimensions and relevance evaluation of the Austrian presidency.

	Political work	Interior politics	Public relations	Reference	Total
Broadsheets					
Observations	804	100	129	382	1415
Mean	0.0844	0.0576	0.0072	−0.0167	0.0482
Minimum	−2.71	−1.542	−4.034	−3.086	−4.034
Maximum	8.828	7.083	5.199	5.886	8.828
Standard deviation	0.9295	1.0354	1.3855	0.9559	0.9939
Tabloids					
Observations	172	19	76	96	363
Mean	0.2378	0.0002	0.3437	−0.0677	0.1128
Minimum	−2.48	−3.393	−1.728	−4.034	−4.034
Maximum	7.169	11.147	6.576	3.719	11.147
Standard deviation	1.2325	1.7771	2.0197	0.7991	1.4269
Total number of observations	976	119	205	478	1778

Compared by type of newspaper, the analysis shows that the presidency is covered more frequently in broadsheets than in tabloids. Distinguishing by frame, the *Reference*, the *Political Work* and the *Interior Politics* frames occur more often in broadsheets, whilst the *Public Relations* frame shows a relative balance. Broadsheets are also more balanced regarding the evaluation of the presidency; tabloids more often discuss the presidency as irrelevant, especially in the dimensions *Public Relations* and *Reference*. The standard deviation is also slightly increased for tabloids, suggesting a higher degree of volatility in the evaluations covered. Regarding the national or regional distribution of newspapers, the analysis shows that coverage is more extensive at the national level.

## Discussion

Arguing that EU Presidencies may in certain configurations contribute to heightened public attention and thus may offer a window of opportunity for EU politics to address the EU's communication and legitimacy deficit, we set out to systematically understand variation in the salience and relevance framing in national media. Looking at eleven years of media coverage of EU presidencies, our analyses suggest that it is the involvement of the national government that contributes to heightened media salience. Resonating with the literature (e.g. Eisele, 2017; Gattermann, 2013) and our hypotheses (*H1a* and *H1b*), the results demonstrate the crucial importance of a national connection for the newsworthiness of EU politics. The Austrian presidency clearly shows the largest effect on salience in the media. However, also the two preceding presidencies forming a Trio with Austria show significant effects that increase in the run-up to the start of the Austrian presidency. This result underscores the argument that the presidency, while arguably having grown less relevant in the decision-making process as such, serves an important role regarding the public's awareness of EU issues and the possible promotion of EU politics among citizens. Presidencies of the own government, thus, can alleviate the EU's communication deficit, potentially helping to better inform European citizens about supranational politics (Marquart et al., 2018) and how it influences what is happening at home (Habermas and Cronin, 2012: 49). Higher media salience may also go beyond an informational value by promoting citizens’ attachment to the supranational polity (e.g. Camaj, 2014).

While effects are small, presidencies of politically weightful member states seem to receive somewhat more attention from Austrian media, in line with *H2a*. In contrast to what we expected, higher public trust in the EU in the country holding the presidency leads to more salience (*H3c*). This is in line with previous literature finding that national newspapers report more about the EU when the domestic public is more supportive, potentially also more interested in EU issues (e.g. Gattermann, 2013). While this is not directly comparable to our analysis, a positive public might also drive more political engagement of its government regarding the priorities of the presidency and the motivation to shape a politically relevant term, thereby also striving more towards drawing public attention to its work.

Our assessment of relevance attributions in the media does not align with our findings for salience. A significant effect is found only for the year 2009: This was the year of the Czech presidency, overshadowed by a heavy political crisis. The Czech government resigned halfway through the presidency (Coman, 2020), which raised doubts about the relevance of the presidency overall and its organisation in the remaining months. This suggests that the specific circumstances of the Czech presidency are an important driver of this effect. Yet, also other presidencies, such as the Greek or Hungarian one, were organised under difficult domestic circumstances and caused severe doubts about the capabilities of the government in question (Coman, 2020). Another explanation could be that the relevance of the presidency in general was discussed in the context of the entering into force of the Lisbon treaty, introducing the permanent President of the EuCo. Overall, the (non-)findings of our relevance analysis may be a mirror of the fact that the success and relevance of presidencies is also depending on the specific political circumstances, like Brexit for example, in which a presidency might be managing rather than actively shaping EU politics.

We additionally provided a more focussed observation regarding the framing of the Austrian presidency. Our analysis showed that interior politics is mostly an issue connected to (a potential) irrelevance of the presidency; it is apparently mostly discussed when (potentially) causing conflict. *Public Relations*, then, attract more attention when they are successful, potentially increasing the relevance of the presidency in terms of publicity. The differences found between broadsheet and tabloid newspapers resonate with the literature as tabloids are usually found to focus more on sensationalism, negativity, and soft news (e.g. Reinemann et al., 2012), and less on hard political news assumed to be the main issue of coverage framing the EU presidency in terms of *Political Work*. The opposite is found for broadsheet newspapers, providing more context and explanation of political issues and processes (e.g. Jandura and Friedrich, 2014). In that sense, it is not surprising that the range of frames of the presidency is higher and evaluations more balanced in broadsheet newspapers, whereas the interest in public relations, around 20% of all articles on the presidency, is higher in tabloids than in broadsheets with below 10% (see Table 5).

These results qualify our more general findings of the longitudinal analysis discussed above: While the general salience of the EU presidency increases when the national government is holding the presidency or is part of a Trio presidency, the focussed analysis shows that more salient coverage is not necessarily promising a more informed debate about EU politics. In contrast, it may just point to influences on or consequences for national politics or party conflicts. In that sense, any optimistic outlook based on heightened media attention due to a country holding the presidency needs to consider that not all news is good news for the EU (see also Marquart et al., 2018). If a presidency shall be strategically used to promote EU politics, it is probably more successful in a context without much political contestation of EU politics.

Concluding, our analysis is, to our best knowledge, the first to conduct a systematic, large-scale analysis of news about the rotating EU presidency. Our study, therefore, addresses a research gap, shedding light on the relevance of the rotating EU presidency in the public eye. The results underline the argument that the rotating EU presidency is a window of opportunity, bringing Europe closer to citizens’ everyday political life. A greater salience of the presidencies held by other member states would be desirable regarding the development of a European public sphere, and the fostering of a political community of Europeans. Yet, the presidency is, to some degree, able to increase the interest of the media and thereby help to somewhat alleviate the communication and legitimacy deficit of the EU.

Future research should, in general, work towards comparing more countries’ coverage to further substantiate the conclusions reached here. It should also include a longer period before 2009 to assess how the introduction of the permanent EuCo President affected coverage overall. Another interesting avenue could be to explore different news outlets and their different audiences. Social media is still a comparably under-researched strand in the study of the EU's legitimacy deficit (but see the Special Issue by de Wilde et al., 2022). In this respect, also a more differentiated analysis of the effects on different age cohorts regarding, for example, the potential to mobilise, would add valuable insights to the debate. We believe our study provides the first step into further investigations.

## Supplemental Material

sj-pdf-1-eup-10.1177_14651165221142504 - Supplemental material for A window of opportunity? The relevance of the rotating European Union presidency in the public eye Click here for additional data file. Supplemental material, sj-pdf-1-eup-10.1177_14651165221142504 for A window of opportunity? The relevance of the rotating European Union presidency in the public eye by Olga Eisele, Tobias Heidenreich, Nina Kriegler, Pamina Syed Ali and Hajo G. Boomgaarden in European Union Politics

sj-R-2-eup-10.1177_14651165221142504 - Supplemental material for A window of opportunity? The relevance of the rotating European Union presidency in the public eye Click here for additional data file. Supplemental material, sj-R-2-eup-10.1177_14651165221142504 for A window of opportunity? The relevance of the rotating European Union presidency in the public eye by Olga Eisele, Tobias Heidenreich, Nina Kriegler, Pamina Syed Ali and Hajo G. Boomgaarden in European Union Politics

sj-R-3-eup-10.1177_14651165221142504 - Supplemental material for A window of opportunity? The relevance of the rotating European Union presidency in the public eye Click here for additional data file. Supplemental material, sj-R-3-eup-10.1177_14651165221142504 for A window of opportunity? The relevance of the rotating European Union presidency in the public eye by Olga Eisele, Tobias Heidenreich, Nina Kriegler, Pamina Syed Ali and Hajo G. Boomgaarden in European Union Politics

sj-R-4-eup-10.1177_14651165221142504 - Supplemental material for A window of opportunity? The relevance of the rotating European Union presidency in the public eye Click here for additional data file. Supplemental material, sj-R-4-eup-10.1177_14651165221142504 for A window of opportunity? The relevance of the rotating European Union presidency in the public eye by Olga Eisele, Tobias Heidenreich, Nina Kriegler, Pamina Syed Ali and Hajo G. Boomgaarden in European Union Politics

sj-R-5-eup-10.1177_14651165221142504 - Supplemental material for A window of opportunity? The relevance of the rotating European Union presidency in the public eye Click here for additional data file. Supplemental material, sj-R-5-eup-10.1177_14651165221142504 for A window of opportunity? The relevance of the rotating European Union presidency in the public eye by Olga Eisele, Tobias Heidenreich, Nina Kriegler, Pamina Syed Ali and Hajo G. Boomgaarden in European Union Politics

sj-csv-6-eup-10.1177_14651165221142504 - Supplemental material for A window of opportunity? The relevance of the rotating European Union presidency in the public eye Click here for additional data file. Supplemental material, sj-csv-6-eup-10.1177_14651165221142504 for A window of opportunity? The relevance of the rotating European Union presidency in the public eye by Olga Eisele, Tobias Heidenreich, Nina Kriegler, Pamina Syed Ali and Hajo G. Boomgaarden in European Union Politics

sj-csv-7-eup-10.1177_14651165221142504 - Supplemental material for A window of opportunity? The relevance of the rotating European Union presidency in the public eye Click here for additional data file. Supplemental material, sj-csv-7-eup-10.1177_14651165221142504 for A window of opportunity? The relevance of the rotating European Union presidency in the public eye by Olga Eisele, Tobias Heidenreich, Nina Kriegler, Pamina Syed Ali and Hajo G. Boomgaarden in European Union Politics

sj-csv-8-eup-10.1177_14651165221142504 - Supplemental material for A window of opportunity? The relevance of the rotating European Union presidency in the public eye Click here for additional data file. Supplemental material, sj-csv-8-eup-10.1177_14651165221142504 for A window of opportunity? The relevance of the rotating European Union presidency in the public eye by Olga Eisele, Tobias Heidenreich, Nina Kriegler, Pamina Syed Ali and Hajo G. Boomgaarden in European Union Politics

sj-yml-9-eup-10.1177_14651165221142504 - Supplemental material for A window of opportunity? The relevance of the rotating European Union presidency in the public eye Click here for additional data file. Supplemental material, sj-yml-9-eup-10.1177_14651165221142504 for A window of opportunity? The relevance of the rotating European Union presidency in the public eye by Olga Eisele, Tobias Heidenreich, Nina Kriegler, Pamina Syed Ali and Hajo G. Boomgaarden in European Union Politics

## Notes

1. The dataset on general EU coverage was retrieved based on an extensive search string. We created an initial list of relevant key terms (e.g. institutions) but also coded key terms inductively on a random sample of articles (*n* = 875). Three coders validated this search string on a fresh sample of 1,500 articles which resulted in satisfactory intercoder reliability (K_alpha_ = 0.86) and validity scores (precision = 0.92, recall = 0.96, F1 = 0.94). The full search string is provided in the Online appendix. The number of media articles on the EU amounts to a total of *n* = 547,617.

2. If not indicated otherwise, information was taken from the europa.eu and Eurostat information pages.

3. All models presented here were also estimated including year fixed effects to account for dynamics over time (leaving out 2018 due to multicollinearity issues with our dummy variable for the AT presidency). The effects for the influence of the AT presidency (*H1a*) were very similar whereas other effects in the salience model were found less robust, especially regarding their *p*-values. Moreover, as indicated in a rather low R-squared, model 2 analysing the influence on relevance evaluation in news coverage is found to have rather low explanatory leverage overall. Not surprisingly, effects found in the model included here were found less robust indicating that research should further investigate possible explanations for how journalists evaluate presidencies in general. Please refer to the Online appendix for further details.

4. Since some outlets did not publish any article on the EU presidency in some months, there is no text data for the scaling approach, resulting in 261 missing cases for this part of the analysis.
