# Supplementary material for: A window of opportunity? The relevance of the rotating European Union presidency in the public eye
Source: Eur Union Polit. 2022 Dec 4;24(2):327–47. doi: 10.1177/14651165221142504 (PMC10189837; doi:10.1177/14651165221142504)
Supplement: sj-pdf-1-eup-10.1177_14651165221142504 - Supplemental material for A window of opportunity? The relevance of the rotating European Union presidency in the public eye [file sj-pdf-1-eup-10.1177_14651165221142504.pdf]

Online Appendix

A window of opportunity? The relevance of the rotating European Union presidency in the public eye

Olga Eisele, Tobias Heidenreich, Nina Kriegler, Pamina Syed Ali & Hajo G. Boomgaarden

Table of Contents

Search string EU. 1

Search string EU presidency. 1

Latent semantic scaling: Political relevance. 1

Additional tables 2

Table A1. Independent variables (country level). 2

Table A2. Coding instructions for ‘relevance’. 5

Table A3. Examples political relevance from 2009-2019 as coded with *LSX*.............. 6

Table A4. Correlation matrix for variables included in linear regressions. 7

Table A5. Variance inflation factors after linear regressions. 8

Table A6. Linear regression models for salience and relevance with individual newspapers. . 9

Table A7. Linear regression models for salience and relevance with individual years. 11

Search string EU.

“eu, europäisch* union, europawahl*, europa-wahl*, europäisch* gerichtshof*, eugh*, europaparlament*, europa-parlament*, europäisch* parlament, europa-spitzenkandidat*, europa-abgeordnete*, europaabgeordnete*, europa-politiker*, europapolitiker*, europäisch* kommiss*, europäisch* zentralbank*, ezb*, eurozone*, euro-zone*, euroland*, euro-land*, euroländer*, euro-länder*, euroraum*, euro-raum*, eurostaat*, euro-staat*, europäisch* gemeinschaftswährung*, europäisch* rat*, rat der eu*, europäisch* ebene, europäisch* rechnungshof*, europäisch* auswärtig* dienst*, europäisch* investitionsbank*, europol*, frontex*, europäisch* agentur*, eurogruppe*, eurorettungsschirm*, europäisch* rettungsschirm, brexit*”

Search string EU presidency.

“'ratsvorsitz*', 'ratspräsidentschaft*', 'eu vorsitz*', 'eu-vorsitz*', 'vorsitz in der eu', 'präsidentschaft im rat‘“

Latent semantic scaling: Political relevance.

Latent Semantic Scaling is an automated content analysis technique providing the means to locate documents on a predefined dimension. Using this technique, we are thus able to create a predefined dimension of “political relevance” of the EU presidency and calculate polarity scores of words. However, as opposed to dictionary methods, these scores are not based on their frequency alone but on their semantic proximity to the chosen seed words. The word-embedding techniques thus provide semantic proximity between words, yet let us choose the basis for this unidimensional scale (Watanabe, 2020). For the variable “political relevance” of the EU presidency, we drew on two different sources for seed words for the two poles (“relevant” vs “not relevant”). First, coders noted markers indicating one of the two poles during the in-depth manual coding for the frames (see section “Manual content analysis” in the manuscript). Second, seed words were chosen on the expert assessment of the authors. Subsequently, a selection of terms was compiled using the most frequent markers from the manual coding in consultation with the results from the expert assessment.

Implemented using the R package LSX, the approach yields a score for each document locating the news items on a fixed scale and allowing for over time comparison (for validation steps, please see section "Automated content analysis" in the manuscript). Example sentences from documents scoring relatively high or low on the "political relevance" scale, respectively, can be found in table A3 in this appendix.

Additional tables

Table A1. Independent variables (country level).

Year	Country	Distance	GDP per capita	Population country	Presidencies before	EU Stance Government	Share EU Critical Parties	Trust in EU
2009	Czech Republic	252.61	14,260	12	0	4.354991	0	48
2009	Sweden	1,241.83	33,840	10	1	6.284140	11.1	45
2010	Spain	1,809.44	23,040	27	3	6.750000	0	43
2010	Belgium	914.88	33,330	12	11	6.523964	0	59
2011	Hungary	214.15	10,250	12	0	5.288676	12.2	54
2011	Poland	558.42	9,860	27	0	6.411388	0	47
2012	Denmark	870.04	45,530	7	6	6.039614	16.2	49
2012	Cyprus	2,013.78	22,500	4	0	6.337996	0	31
2013	Ireland	1,681.13	38,830	7	6	6.262774	2.4	29
2013	Lithuania	947.36	11,850	7	0	5.433633	0	52
2014	Greece	1,282.84	16,270	12	4	6.555555	16.7	24

2014	Italy	764.80	26,980	29	11	6.592622	11.9	29
2015	Latvia	1,101.61	12,430	4	0	6.332500	0	51
2015	Luxemburg	763.96	95,090	4	11	6.333333	0	45
2016	Netherlands	936.51	41,590	13	11	5.312997	20.0	40
2016	Slovakia	54.88	14,920	7	0	6.142857	0	42
2017	Malta	1,377.22	25,530	3	0	6.142857	0	56
2017	Estonia	1,361.71	18,120	4	0	5.368129	18.8	49
2018	Bulgaria	817.47	8,000	10	0	5.972206	0	56
2018	Austria	0.00	43,610	10	2	4.180790	16.9	45
2019	Romania	566.35	11,520	14	0	4.725203	0.0	52
2019	Finland	1,439.20	43,480	7	2	5.849614	19.7	56

Coding instructions for ‘relevance’

Definition: Variable to define if the presidency is evaluated as relevant or not. Please decide what is your overall impression based on the whole article (i.e., not only one frame).

Coding Rules: To code this variable, please read the article and pay attention to the following questions. Is the presidency described as

- ... politically strong and powerful?
- ... 'having done a good job'?
- ... having succeeded in, e.g., uniting member states or getting a proposal accepted?
- ... having had a strong influence on EU politics?
- ... being very organised and efficient?
- ... having experts on board or exhibiting strong expertise?
- ... having mastered the challenges defined beforehand? Or having fulfilled what it promised? Lived up to the priorities formulated beforehand?
- ... giving their guests a good time (e.g., when public relations was coded); e.g., good food, nice location, 'Höhepunkt', etc.
- ... working for the EU instead of only trying to push national interests?
- ... having mastered an intense workload?
- ... behaving in a way that is adequate for an EU presidency? (acting as an example; ‘Vorbildrolle’)

If the answer to these questions is yes, code yes. If it is not, then code no. Please only code yes/no if you find that there is an EXPLICIT evaluation, i.e., a clear answer to the questions formulated above. Also remember that the evaluation MUST ALWAYS be related to the presidency itself or people described as representing it; i.e., not the EU itself or the Austrian parliament or some other actor that appears in the text. If you cannot answer the questions formulated above with a clear yes or no (for the whole article), then code neutral/ambivalent.

Examples given in the codebook (only for illustration; note that we only coded this for the Austrian presidency in this (manually coded) part of the analysis).

Table A2. Coding instructions for 'relevance'.

irrelevant	The European Union has not failed completely in the crisis, but it has largely failed. This applies not only to the European Commission under Ursula von der Leyen. It also applies to the Council Presidency, especially the German Presidency under Chancellor Angela Merkel in the second half of 2020.<br><br>The Slovenian government, which holds the Council presidency, even wanted to write the accession date of 2030 into the summit conclusions for the first time, which did not find a majority.
neutral/ ambivalent	On Tuesday, the Slovenian EU presidency cancelled preparatory talks for a U.S.-EU summit on trade and technology policy - in the first tangible sign of support for member country France.<br><br>"For a successful digital transformation and the achievement of our climate goals, we need data-driven innovation built on the availability of data," said Slovenia's Minister of Public Administration, Boštjan Koritnik, speaking on behalf of the Slovenian EU Presidency<br><br>EU Commission chief Jean-Claude Juncker described the Austrian EU presidency's record as "impressive in every respect." 134 dossiers had been successfully dealt with. He said he would like to see more presidencies with such stringency. There was only one exception, Juncker stressed. He would have liked Austria to have agreed to the UN migration pact "instead of sending out negative signals." He could not understand this decision, but Austria was also not the only country that had made such a decision.
relevant	Chancellor Sebastian Kurz (ÖVP) on Tuesday gave a positive summary of Austria's EU Council presidency. He said the country had succeeded in moving the EU forward. Austria will continue to contribute to strengthening the EU, Kurz told Members of the European Parliament in Strasbourg.

Table A3. Examples political relevance from 2009-2019 as coded with *LSX*.

**Political Relevance**	**Example Sentence(s): English Translation**	**Example Sentence(s): German original**
Low	Two days before the start of Hungary's EU Council Presidency, the national-conservative leadership in Budapest has thus created a fait accompli. The law is seen by critics, including EU leaders, as a massive attack on press freedom.	Zwei Tage vor dem Beginn der ungarischen EU-Ratspräsidentschaft schafft die national-konservative Führung in Budapest damit vollendete Tatsachen. Das Gesetz wird von Kritikern, darunter EU-Spitzenpolitikern, als massiver Angriff auf die Pressefreiheit gesehen.
	Austria's Chancellor Sebastian Kurz has not managed to fulfill his self-chosen role as "bridge builder" in Europe in the past six months. At the beginning of the EU Presidency, he also talked a little too much and raised expectations that were too high.	Österreichs Kanzler Sebastian Kurz hat es in den vergangenen sechs Monaten nicht geschafft, seine selbst gewählte Rolle als "Brückenbauer" in Europa auszufüllen. Zu Beginn des EU-Vorsitzes hatte er auch den Mund etwas zu voll genommen und zu hohe Erwartungen geweckt.
	When asked by a journalist whether this was a sign of the new Europe, Czech Prime Minister and EU Council President Mirek Topolanek responded with a pointed comment.	Auf die Frage einer Journalistin, ob dies ein Zeichen für das neue Europa sei, reagierte der tschechische Premier und EU-Ratschef Mirek Topolanek mit der spitzen Bemerkung.
High	Kurz: "Our goal is to make a positive contribution to the further development of the European Union, especially during the Austrian EU Council Presidency in the second half of the year."	Kurz: „Unser Ziel ist es, einen positiven Beitrag zur Weiterentwicklung der Europäischen Union zu leisten, insbesondere während des österreichischen EU-Ratsvorsitzes in der zweiten Jahreshälfte."
	It is the last highlight of the Austrian EU Presidency: On Tuesday, the EU-Africa Summit will take place in the Austria Center. This is intended to give new impetus to the economic relations between the two continents.	Es ist der letzte Höhepunkt des österreichischen EU-Vorsitzes: Am Dienstag geht im Austria Center der EU-Afrika-Gipfel über die Bühne. Damit soll neuer Schwung in die wirtschaftlichen Beziehungen zwischen den beiden Kontinenten gebracht werden.
	Austria presented itself at the EU conference against anti-Semitism as a pioneering country. Federal Chancellor Sebastian Kurz emphasized that as the EU Presidency, anti-Zionism was also a conscious topic at the conference.	Österreich präsentierte sich bei der EU-Konferenz gegen Antisemitismus als Vorreiterland. Bundeskanzler Sebastian Kurz betonte, dass man als EU-Vorsitzland bewusst bei der Konferenz auch Antizionismus zum Thema gemacht habe.

Table A4. Correlation matrix for variables included in linear regressions.

	Bulgaria	Estonia	Distance	Population country	GDP	Presidencies before	Government EU Stance	Share critical parties	Trust in EU
Austria	-.05	-.05	-.4	-.05	.19	-.06	-.53	.28	-.01
Bulgaria		-.05	-.06	-.08	-.23	-.17	.03	-.19	.24
Estonia			.17	-.16	-.11	-.17	-.16	.33	.08
Distance				-.06	.08	.05	.55	-.01	-.27
Population country					-.16	.29	.24	.01	-.3
GDP						.66	.1	.22	-.1
Presidencies before							.29	.23	-.3
Government EU Stance								-.23	-.29
Share critical parties									-.17

Notes. Pearson correlation coefficients.

Table A5. Variance inflation factors after linear regressions.

	Austria	Bulgaria	Estonia	Distance	Population country	GDP	Presidencies before	Government EU Stance	Share critical parties	Trust in EU
Model: DV Salience	2.07	1.18	1.38	1.87	1.68	3.09	3.34	2.62	1.52	1.49
Model: DV Relevance	2.26	1.23	1.42	1.97	1.63	3.13	3.20	2.74	1.56	1.46

Table A6. Linear regression models for salience and relevance with individual newspapers.

	Model 1: Salience B (SE)	Model 2: Relevance B (SE)
Hypothesis 1		
Austria	.654*** (.023)	.005 (.038)
Trio presidency 2: Bulgaria	.430*** (.017)	.037 (.028)
Trio presidency 1: Estonia	.073*** (.019)	-.009 (.032)
Distance	.033 (.022)	-.050 (.039)
Hypothesis 2		
Population country	.068*** (.020)	-.017 (.037)
GDP	.001 (.028)	.085+ (.050)
Presidencies before	-.004 (.029)	-.089+ (.051)
Hypothesis 3		
Government EU Stance	-.030 (.025)	.050 (.044)
Share critical parties	-.032+ (.019)	.031 (.035)
Trust in EU	.064*** (.019)	-.033 (.035)
Controls		
Year 2009	.222*** (.018)	-.166*** (.030)
Die Presse (vs. Der Standard)	-.168* (.075)	-.045 (.122)
Heute (vs. Der Standard)	-.366*** (.075)	.048 (.160)
Kleine Zeitung (vs. Der Standard)	-.327*** (.075)	-.023 (.125)
Kronenzeitung (vs. Der Standard)	-.480*** (.075)	-.217 (.135)
Kurier (vs. Der Standard)	-.126+ (.075)	-.140 (.123)
Oberösterreichische Nachrichten (vs. Der Standard)	-.256*** (.075)	-.083 (.129)
Österreich (vs. Der Standard)	.085 (.094)	.081 (.186)

Salzburger Nachrichten (vs. Der Standard)	-.096<br>(.075)	.053<br>(.125)
Tiroler Zeitung (vs. Der Standard)	-.157*<br>(.075)	.138<br>(.126)
Vorarlberger Nachrichten (vs. Der Standard)	-.229**<br>(.075)	.099<br>(.130)
Wiener Zeitung (vs. Der Standard)	.107<br>(.075)	.046<br>(.123)
R2	.628	.031
F	F (22,1492) = 117<br>p < .05	F (22,1231) = 2.8<br>p < .05
N	1,515	1,254

Notes: Coefficients are standardised B-coefficients, standard errors in parentheses; +p < .1, *p < .05, **p < .01, ***p < .001.

Table A7. Linear regression models for salience and relevance with individual years.

	Model 1: Salience<br>B (SE)	Model 2: Relevance<br>B (SE)
Hypothesis 1		
Austria	.416***<br>(.033)	.099+<br>(.056)
Trio Presidency 2: Bulgaria	.329***<br>(.027)	.179***<br>(.045)
Trio Presidency 1: Estonia	-.022<br>(.039)	-.092<br>(.068)
Distance	-.121*<br>(.059)	-.296**<br>(.107)
Hypothesis 2		
Population country	.034<br>(.026)	-.031<br>(.046)
GDP	.003 (.047)	-.070<br>(.086)
Presidencies before	-.042<br>(.045)	-.001<br>(.080)
Hypothesis 3		
Government EU Stance	-.025<br>(.045)	.223**<br>(.068)
Share critical parties	.066<br>(.048)	.170*<br>(.086)
Trust in EU	-.139*<br>(.048)	-.247*<br>(.116)
Controls		
Year 2010 (vs. Year 2009)	-.239<br>(.158)	.600*<br>(.272)
Year 2011 (vs. Year 2009)	-.754***<br>(.102)	.161<br>(.178)
Year 2012 (vs. Year 2009)	-.721***<br>(.092)	.356*<br>(.156)
Year 2013 (vs. Year 2009)	-.754***<br>(.100)	.749***<br>(.177)
Year 2014 (vs. Year 2009)	-1.154***<br>(.223)	-.234<br>(.410)
Year 2015 (vs. Year 2009)	-.724***<br>(.120)	.720***<br>(.215)
Year 2016 (vs. Year 2009)	-.920***<br>(.129)	.020<br>(.232)

Year 2017 (vs. Year 2009)	-.260<br>(.170)	1.048***<br>(.300)
Year 2019 (vs. Year 2009)	-.577***<br>(.170)	1.017***<br>(.166)
Outlet type: broadsheet (vs. tabloid)	.089***<br>(.075)	.012<br>(.037)
Level: national (vs. regional)	.031<br>(.011)	-.049<br>(.031)
R2	.615	.042
F	F (21,1493) = 116<br>p < .05	F (21,1232) = 3.609<br>p < .05
N	1,515	1,254

Notes: Coefficients are standardised B-coefficients, standard errors in parentheses; +p < .1, *p < .05, **p < .01, ***p < .001.
